# Serum CEACAM1 Correlates with Disease Progression and Survival in Malignant Melanoma Patients

**DOI:** 10.1155/2012/290536

**Published:** 2012-01-16

**Authors:** Sapoznik Sivan, Faranesh Suzan, Ortenberg Rona, Hamburger Tamar, Barak Vivian, Peretz Tamar, Schachter Jacob, Markel Gal, Lotem Michal

**Affiliations:** ^1^The Ella Institute for Treatment and Research of Melanoma and Skin Cancer, The Sheba Cancer Research Center, The chaim Sheba Medical Center, Tel Hashomer 52621, Israel; ^2^Sharett Institute of Oncology, Hadassah Medical Center, Jerusalem, Israel; ^3^Department of Clinical Microbiology and Immunology, Sackler Faculty of Medicine, Tel Aviv University, Tel Aviv, Israel; ^4^Talpiot Medical Leadership Program, The chaim Sheba Medical Center, Tel Hashomer, Israel

## Abstract

The search for melanoma biomarkers is crucial, as the incidence of melanoma continues to rise. We have previously demonstrated that serum CEACAM1 (sCEACAM1) is secreted from melanoma cells and correlates with disease progression in metastatic melanoma patients. Here, we have used a different cohort of melanoma patients with regional or metastatic disease (*N* = 49), treated with autologous vaccination. By monitoring sCEACAM1 in serum samples obtained prior to and after vaccination, we show that sCEACAM1 correlates with disease state, overall survival, and S100B. The trend of change in sCEACAM1 following vaccination (increase/decrease) inversely correlates with overall survival. DTH skin test is used to evaluate patients' anti-melanoma immune response and to predict response to vaccination. Importantly, sCEACAM1 had a stronger prognostic value than that of DTH, and when sCEACAM1 decreased following treatment, this was the dominant predictor of increased survival. Collectively, our results point out the relevance of sCEACAM1 in monitoring melanoma patients.

## 1. Introduction

Malignant melanoma is a main cancer-related cause of death in people below 30. While its incidence continues to rise more rapidly than that of any other malignancy, until lately, therapy had shown only moderate success and caused numerous adverse effects [1–3]. A new hope for melanoma patients has emerged now from the development of a specific B-RAF inhibitor and the entry of immune checkpoint modulators to the clinic. In spite of this progress, the monitoring of melanoma patients still presents a clinical challenge as it heavily relies on history taking, physical examination, and wide imaging studies [4]. This, together with the fact that melanoma can remain dormant for long periods of time before relapsing [5], emphasizes the need for valid melanoma biomarkers. Currently, the two most widely used melanoma biomarkers are lactate dehydrogenase (LDH) and the calcium binding protein S100B [6–8]. Serum levels of S100B or LDH correlate with poor outcome and are associated with shorter disease-free and overall survival [9, 10]. Several studies showed the prognostic value of S100B and LDH in predicting successful therapeutic treatments for malignant melanoma patients [11–16]. Unfortunately, however, serum S100B and LDH are not specific for melanoma. Abnormal elevation of S100B accompanies liver and kidney injuries as well as inflammatory and infectious diseases [17], while elevated LDH is also observed in liver injury, cell damage, hemolysis, and so forth [18–20].

CEACAM1 (carcinoembryonic antigen-related cell adhesion molecule 1) is a transmembrane multifunctional cell-cell adhesion molecule, belonging to CEACAM, a subdivision of the Ig Superfamily. Broadly expressed in human epithelial, endothelial, and hematopoietic cells, it regulates immune responses, neovascularization, and insulin clearance (reviewed in [21, 22]). Membranal CEACAM1 (mCEACAM1) expression is downregulated in some types of cancer [23–26] and its reexpression by tumor cells inhibits *in vivo* tumor growth [27, 28], leading to the original definition of mCEACAM1 as a tumor suppressor. However, in several cancers, including malignant melanoma and non-small-cell lung cancer, mCEACAM1 is upregulated and its expression highly correlates with tumor progression, the development of metastasis, and poor survival [29–31]. Immunohistochemical analysis on superficial spreading melanoma, dysplastic nevi and benign nevi, showed that mCEACAM1 is stepwise elevated during the course of malignant melanoma progression [32]. Patient monitoring proved that its predictive value for metastasis formation and poor survival is superior to that of tumor thickness and independent of other factors, including ulceration, tumor thickness, and mitotic rate [29]. Mechanistic evidence regarding the role of mCEACAM1 in melanoma is scarce. *In vitro* studies have demonstrated that mCEACAM1 promotes melanoma cell migration and invasion [33] as well as protection from elimination by cytotoxic NK and T cells [34–36]. We have recently identified a soluble form of human CEACAM1 (sCEACAM1), which is produced and secreted from melanoma cells in a process that demands active protein synthesis and intact intracellular vesicular transport [37]. Monitoring of metastatic melanoma patients for serum levels of sCEACAM1 showed that patients with evidence of disease (WED) exhibit significantly higher serum sCEACAM1 levels as compared to patients with no clinical evidence of disease (NED) or with healthy volunteers. sCEACAM1 levels correlated with LDH, and most importantly, stratified the metastatic patients into two prognostic groups with different survival rates [37]. These results exhibit the prognostic value of sCEACAM1 for melanoma progression and survival.

In this study, we monitored melanoma patients with regional or metastatic disease, treated with autologous cell vaccination. Melanoma is unique among human cancers as it induces significant numbers of anti-tumor reactive lymphocytes during the natural course of tumor growth [38]. Vaccination with modified autologous melanoma cells given as a postsurgical adjuvant therapy is thought to elicit this naturally occurring immune response and to prolong disease-free period [39, 40]. Vaccination may be beneficial especially in selected patients who show successful anti-melanoma immune response, as reflected by the delayed-type-hypersensitivity (DTH) test (i.e., positive skin reaction to subcutaneous injection of unmodified autologous melanoma cells) [39, 40].

Here, we monitored 49 melanoma patients (AJCC stages III-IV) treated with autologous tumor vaccination in the years 1998–2010 and focused on sCEACAM1 evaluation. We found that sCEACAM1 correlates with disease state and is also likely to correlate with survival rate. Moreover, the change in sCEACAM1 over time (increase or decrease) correlated with overall survival and had a superior value over DTH skin response. In addition, post-vaccination sCEACAM1 correlated with S100B. These observations support the prognostic value of sCECACM1 and its potential role in monitoring of melanoma patients with regional or metastatic disease.

## 2. Patients and Methods

### 2.1. Patients

Patients with pathologically verified cutaneous MM in AJCC stages III-IV in the years 1998–2010 were included. Two patients with thick cutaneous melanomas AJCC stage IIB were included in this series, on a compassionate basis. Clinical characteristics of participants are detailed in Table 1. There were no exclusion criteria. All NED patients were treatment-naïve (were not treated before vaccination). WED patients were accrued on the condition that they had progressed following first-line treatment (DTIC, IL-2 or both). Patients' evaluation was done by CT scan of the whole body, performed within 28 days prior to treatment initiation. All melanoma patients gave written informed consent prior to their participation in this study. This study was approved by the Institutional Review Board of Hadassah Hebrew University Hospital, Jerusalem.

### 2.2. Vaccination

The protocol used for vaccine preparation and delivery was as previously described [40]. Both NED and WED patients were treated with the same protocol. Briefly, 10–25 × 10^6^ autologous melanoma cells were subcutaneously injected in each dose of vaccine. On treatment day, the cells were thawed, washed, and irradiated to 170 Gy. Conjugation of melanoma cells with DNP (dinitrophenol) was performed by the method of Miller and Claman [41]. Bacille Calmete Guerin (BCG) was used as an adjuvant and mixed with tumor cells. DNP sensitization was induced by applying 0.1 mL of 2% DNP dissolved in acetone-corn oil (Sigma Aldrich) topically to the inner aspect of the arm. The first two vaccine doses were preceded by cyclophosphamide, 300 mg/m^2^, given as an immunomodulatory dose. The vaccine was injected into 3 adjacent sites on the upper arm or thigh, avoiding limbs where lymph node dissection has been previously performed. An overall of eight doses of vaccine were administrated at intervals of 21–28 days. 

### 2.3. Specimen Characteristics

Blood samples were obtained from patients before the first vaccine was administered (usually up to 2 months after surgery), and following the 5th or 8th vaccination, by venipuncture and standard handling procedures. 15 milliliters of blood were collected in citrate-containing tubes (BD Biosciences) and then centrifuged at 700 g for 10 minutes in room temperature to obtain sera. All serum samples were collected and divided into aliquots and frozen in −80°C until analysis. Anonymous samples (marked only with ID number) were linked only to clinical-pathological data. 

### 2.4. CEACAM1 and S100B Evaluation by ELISA

**Figure 1:**
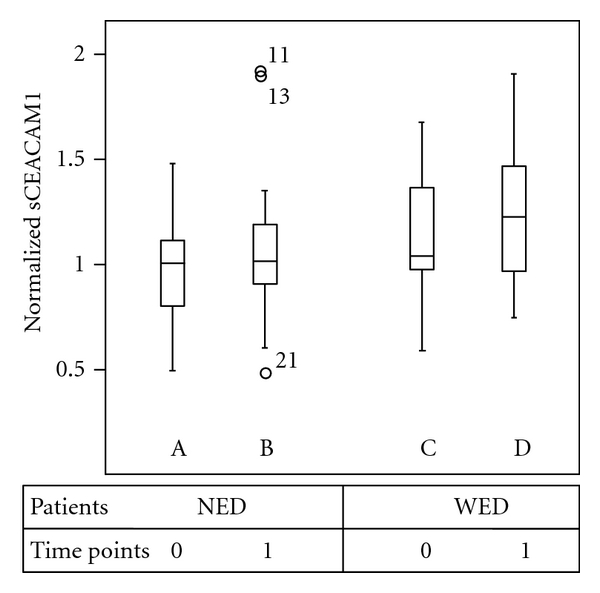
sCEACAM1 in WED patients increases over time and as compared with NED patients. sCEACAM1 was measured by ELISA in serum samples of 29 NED (A, B) and 20 WED (C, D) malignant melanoma patients, both before (time point 0) and following treatment (time point 1). Vertical lines indicate medians.

**Figure 2:**
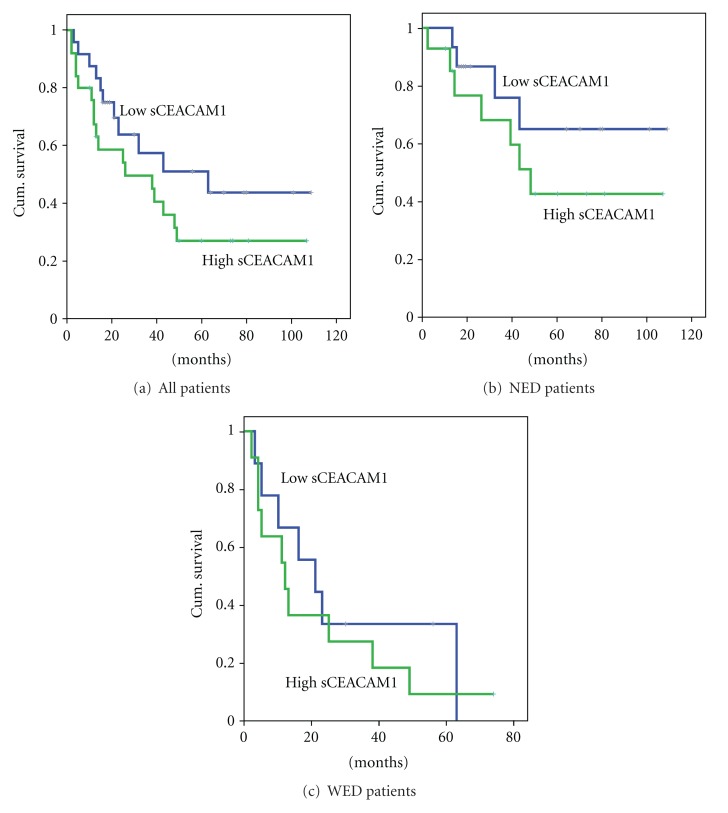
The correlations between sCEACAM1 and survival. The whole group of patients and NED exclusively or WED exclusively were divided into two groups (“low”/“high”) according to pre-treatment sCEACAM1 median level, and the survival rate of each subgroup was analyzed by Kaplan-Meier analysis. Group sizes were as follows: *N* = 24 (low) and *N* = 25 (high) in (a); *N* = 15 (low) and *N* = 14 (high) in (b).

**Figure 3:**
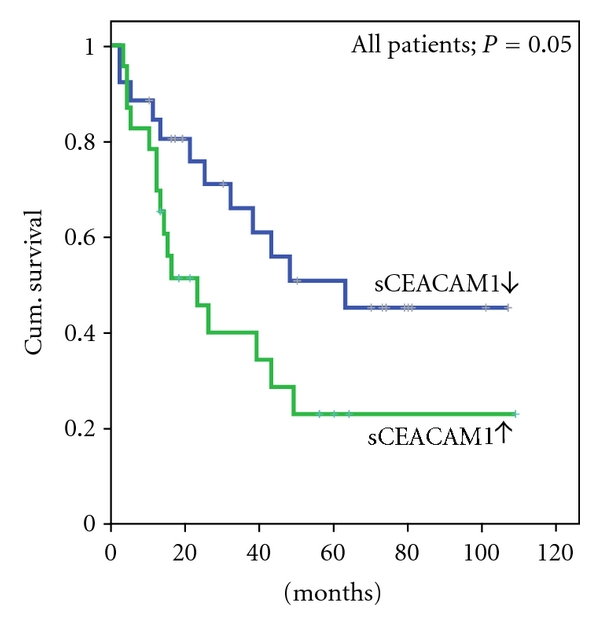
The change of sCEACAM1 following vaccination inversely correlates with survival. The change in sCEACAM1 following treatment (post minus pre-vaccination) was calculated and the patients (*N* = 49) were divided according to the trend of sCEACAM1 change. Kaplan-Meier plots were used to describe the survival rates of each subgroup of patients. Groups sizes were as follows: *N* = 26 (decreased sCEACAM1) and *N* = 23 (increased sCEACAM1).

**Figure 4:**
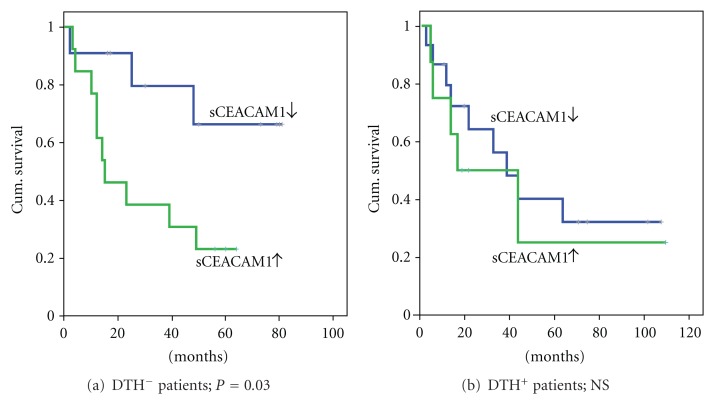
Monitoring of sCEACAM1 further stratifies DTH-negative patients into two prognostically distinct groups. The analysis described in Figure 3 was performed exclusively for (a) DTH-negative patients (*N* = 24) and (b) DTH-positive patients (*N* = 23). Subgroups sizes were *N* = 11 (DTH^−^) or *N* = 15 (DTH^+^) for decreased sCEACAM1 and *N* = 13 (DTH^−^) or *N* = 8 (DTH^+^) for increased sCEACAM1.

sCEACAM1 serum levels were measured by the Sandwich ELISA protocol described in [37]. Soluble S100B in the serum was estimated by ELISA according to the manufacturer's instruction [42]. Half of the samples were analyzed in Hadassah Medical Center and the others in Sheba Medical Center. The results obtained from the two medical institutes showed some differences, probably due to variability in sample handling, freezing/thawing cycles, and batches of antibodies used. In order to compensate for these differences, the two medians (one for each group of samples) were calculated, and each sCEACAM1 value was divided by the median of its group (Figures 1, 2 and Table 2). In this analysis, sCEACAM1 values which equal the median are represented by 1 and values high/lower than median by >1 or <1 values. Similar normalization was performed for S100B (Table 2). When analyzing ΔsCEACAM1 values (post-vaccinations 5th or 8th minus pre-1st vaccination levels, Figures 3-4), absolute rather than normalized sCEACAM1 values were used. 

### 2.5. DTH Evaluation

Skin testing to evaluate delayed type-hypersensitivity to autologous melanoma cells was performed by intradermal injection of 1–3 × 10^6^ unmodified melanoma cells irradiated at a dose of 170 Gy, as already described in [40]. We arbitrarily chose the value of 10 mm of erythema to discriminate between negative DTH (<10 mm) and positive DTH (≥10 mm). 

### 2.6. Study Design

This study was retrospective. No stratification or matching were used and patients (the great majority of them from AJCC stages III and IV) were selected in a random manner. Sample size (*N* = 49) matched previous similar studies (reviewed in [43]) and was sufficient for analysis of the results. Samples were obtained from June 1998 through October 2010. Median follow-up time was 23 months. 

### 2.7. Statistical Analysis

The analysis was focused on the impact of sCEACAM1 on disease progression and survival. Overall survival was estimated by the Kaplan-Meier's method. Significance (*P* value) was calculated by Mantel-Cox regression. 

## 3. Results

### 3.1. Soluble CEACAM1 Correlates with Disease State

Our study encompassed 49 melanoma patients, staged, based on AJCC 2002, as AJCC II (*N* = 2), III (*N* = 32), and IV (*N* = 15), that were treated with autologous vaccination (Table 1). The patients were categorized according to the clinical manifestation of disease into patients with no evidence of disease (NED; *N* = 29; 22/29 in AJCC III) and patients with active disease (WED; *N* = 20, 10/20 in AJCC III and 10/20 in AJCC IV). Accordingly, most patients exhibited normal LDH values (Table 1). It should be noted that autologous vaccination is beneficial for selected patients and uncommonly yields objective tumor regressions [39, 40]. In our cohort of patients, it did not result in any tumor regression (Table 1). Measurement of serum CEACAM1 (sCEACAM1) in blood samples over time (i.e., before as compared to following vaccination; Figure 1, C as compared to D), revealed a 20% elevation of sCEACAM1 in the WED group and no elevation in the NED group (A as compared to B). Comparison of WED to NED patients demonstrated a 20% elevation in WED patients, both at basal time point (Figure 1, mean sCEACAM1 = 0.93 in a as compared to 1.11 in C; *P* = 0.024) and following vaccination (Figure 1, mean sCEACAM1 = 1.0 in B as compared to 1.19 in D; *P* = 0.068). These results are in line with our previous findings in a different cohort of melanoma patients and treatments, describing a significant elevation in sCEACAM1 in WED as compared to NED patients and healthy volunteers [37]. 

### 3.2. Soluble CEACAM1 Correlates with Survival in NED Patients

We next categorized the whole group of patients according to their basal (pre-treatment) sCEACAM1 values into “high” and “low” subgroups (see *“Methods”*). Analysis of overall survival rates using Kaplan-Meier plots revealed an inverse correlation between sCEACAM1 and survival (Figure 2). This correlation was evident though it did not reach statistical significance. While in the low-sCEACAM1 subgroup (Figure 2(a); black, *N* = 24), the mean overall survival rate was 62 months, it was only 44 months for high-sCEACAM1 patients (Figure 2(a); gray, *N* = 25) and 49 months for the whole population of patients. In order to rule out the possibility that these results stem from the fact that most low-sCEACAM1 patients (70.8%) were NED (i.e., patients whose expected survival is higher), the same analysis was performed for each of the patients groups separately. As can be seen in Figure 2(b), NED patients whose sCEACAM1 was low were likely to have a higher overall survival rate (black, 80.8 months, *N* = 15) as compared to sCEACAM1^high^ NED patients (Gray, 61 months, *N* = 14). In WED patients, pre-treatment sCEACAM1 had no prognostic value on survival rate (Figure 2(c)).

### 3.3. The Change in sCEACAM1 over Time Inversely Correlates with Survival

In order to test the correlation between sCEACAM1 and survival in the whole group, independently of patients' status as NED or WED, we calculated the change in sCEACAM1 after treatment for each of the 49 patients (ΔsCEACAM1). The sCEACAM1 levels before treatment served as the point of reference. Patients were divided into two groups according to the trend (“increased”/“decreased”) of ΔsCEACAM1 and Kaplan-Meier analysis was performed for each of the groups (Figure 3). Remarkably, the 26 patients that exhibited a decrease in sCEACAM1 levels during followup were characterized by a mean overall survival rate of 63 months, whereas the 23 patients in which sCEACAM1 was increased had a mean survival rate of only 40 months (*P* = 0.055). The trend of change of sCEACAM1 thus positively correlated with survival. 

### 3.4. The Correlation of ΔsCEACAM1 with Survival Is Stronger than That of DTH Test

DTH (delayed-type hypersensitivity) skin reactivity using unmodified autologous melanoma cells is used to predict the ability of patients to develop an immune response against his/her tumor and is attributed to the vaccination procedure [39, 40]. Survival rate of the patients was analyzed in DTH-negative and DTH-positive groups, according to the trend in ΔsCEACAM1. Surprisingly, the DTH-negative group, that is, patients that were not expected to gain a survival benefit from the vaccine (*N* = 24), could be categorized according to ΔsCEACAM1 into two distinct prognostic groups (Figure 4(a)). Indeed, the 11 patients that exhibited a decrease in sCEACAM1 had a mean overall survival rate of 63 months, as compared to only 29 months in the 13 patients in which sCEACAM1 levels were increased (Figure 4, *P* = 0.03). In contrast, no significant differences were found between ΔsCEACAM1 subgroups in DTH-positive patients (*P* = 0.58, Figure 4(b)). These results indicate that CEACAM1 monitoring with ΔsCEACAM1 has an added and complimentary value to the DTH response test. 

### 3.5. The Correlation Between sCEACAM1 and S100B

We have previously demonstrated that sCEACAM1 significantly correlates with LDH serum levels in metastatic melanoma patients [37]. Here, we analyzed the correlation between sCEACAM1 and another known melanoma serum biomarker, S100B. We could not observe a correlation between the absolute values of these two factors (data not shown). However, when categorizing values into high/low subgroups, we found a significant (*P* = 0.02) correlation between post-vaccination S100B and sCEACAM1 (Table 2), that is, low sCEACAM1 was most likely to be accompanied by low S100B in the same patient, and vice versa. To conclude, sCEACAM1 correlated with disease state and with S100B and its dynamics over time highly correlated with overall survival rates.

## 4. Discussion

In this retrospective study, we examined 49 melanoma patients from advanced AJCC stages, before and following autologous vaccination, for serum sCEACAM1 levels. We found that sCEACAM1 increases over time in WED patients and that their sCEACAM1 levels are higher as compared to NED patients (Figure 1). This confirms our previously published results with a different cohort of melanoma patients and treatments [37]. As most NED and WED patients are at AJCC Stages III and IV, respectively, this result implies that sCEACAM1 could reflect disease burden. Indeed, sCEACAM1 is synthesized and secreted from cultured human melanoma cells and its concentrations correlate with the amount of seeded melanoma cells *in vitro* [37] as well as with tumor mass in mice (unpublished data). In addition, post-vaccination sCEACAM1 correlates with S100B (Table 2), which sensitively reflects tumor mass [9]. Collectively, this data further fortifies the possible value of sCEACAM1 in monitoring disease burden. 

Importantly, high sCEACAM1 levels are likely to correlate with poor overall survival (Figure 2) and significantly discriminated between patients who died and patients who remained alive during follow-ups. However, to rule out the possibility that these observations stem entirely from the correlation of sCEACAM levels with disease burden (stage and evidence of disease) we analyzed the trend of sCEACAM1 change over time (post-vaccination minus pre-vaccination) for each of the patients. Remarkably, “increased sCEACAM1” patients had significantly poor overall survival rates as compared with “decreased sCEACAM1” patients, both in the whole group of patients (Figure 3) and in NED or WED patients subgroups. This indicates that monitoring of serum sCEACAM1 in melanoma patients has a prognostic predictive value. Furthermore, the majority of the patients in this cohort exhibited normal values of serum LDH (Table 1). This was not surprising, as the majority of the patients were either with no evidence of disease (29/49 patients) or with Stage III (10/49 patients). Blood marker levels were compared 6-7 m in average after initiation of treatment. None of the NED patients and most Stage III patients exhibited clinically evident progression during this period, which was supported by the normal LDH values. In contrast, sCEACAM1 levels enabled predictive stratification of the patients (Figures 2 and 3). It is therefore implied that in patients with normal LDH (mainly NED and Stage III), sCEACAM1 might have superior predictive value. 

We have previously reported that autologous vaccination was associated with improved overall and disease-free survival in AJCC stage III melanoma patients who attained strong skin reactivity against their tumor cells [39, 40]. The decrease in sCEACAM1 in NED/Stage III patients following vaccination is in line with this data. Moreover, more “decreased sCEACAM1” patients were found among DTH-positive patients (15/23 = 65%) as compared with DTH-negative patients (11/24 = 46%), but this difference did not reach statistical significance, probably due to small population size. Interestingly, monitoring of ΔsCEACAM1 further identified two distinct prognostic subgroups (*P* = 0.03) among the DTH-negative patients, but not in the DTH-positive patients. It is implied that the change in sCEACAM1 during vaccination can identify more subtle, yet of prognostic importance, immune events that the crud skin test is unable to show. Therefore, sCEACAM1 has an added prognostic value to DTH test and both could be used in adjunct to achieve superior patient stratification. 

Overall, the current data suggest that the alterations in serum sCEACAM1 levels in melanoma patients reflect disease activity and support its role as a reliable serum marker. This prognostic value could be derived from the reflection of disease burden by sCEACAM1, as described previously [37]. However, serum sCEACAM1 may be more than a biomarker and may also have a biological functional and play an active role in facilitating melanoma aggressiveness. The previous findings that sCEACAM1 is produced by active protein synthesis in melanoma cells and that its production does not result from protein cleavage [37] actually support this idea. Membrane-bound CEACAM1 protects melanoma cells from NK and T cells-mediated cytotoxicity and enable them to avoid immune attack. Expressed and secreted from melanoma cells but not from immune cells [37], sCEACAM1 might act in a similar manner as a soluble agonistic ligand, which activates membrane-bound CEACAM1 receptors on NK and T cells thereby inhibiting their effector functions. sCEACAM1 may also agonistically enhance other CEACAM1-mediated functions, such as angiogenesis. Alternatively, it antagonize membrane-bound CEACAM1 to inhibit the adhesive interactions between lymphocytes and activated endothelial cells, thus affecting the rolling, adhesion, and recruitment of lymphocytes. These hypotheses remain to be proven in future investigations. 

The mechanism of sCEACAM1 production is currently unknown. It was shown in mice that removal of Exon 4 by alternative splicing generates a truncated protein due to a stop codon created at the junction between Exon 3 and Exon 5 [44]. A similar sequence analysis of the human *CEACAM1* shows that the junction between Exons 3 and 5 creates a new stop codon, thus sCEACAM1 may be formed as a result of specific alternative splicing. Revealing the cues that induce sCEACAM1 expression/secretion, as well as characterization of sCEACAM1 functional domains, will help in deciphering whether the intriguing sCEACAM1 protein harbors biological functionality. 

## Figures and Tables

**Table 1:** Depiction of the clinical characteristics of 20 WED and 29 NED melanoma patients used in this study.

Total number of patients	49 (100%)		
*Age (years) at treatment*		*Sex, Female*	25 (51%)
<40	9 (18.4%)		
41–60	16 (32.7%)		
>61	24 (49%)		

*Stage at presentation*		*Time from first treatment to last follow up date *	
Stage II	2	0–12 months	12 (24.5%)
Stage III	32	13–24 months	13 (26.5%)
Stage IV	15	25–36 months	4 (8.2%)
		>37 months	20 (40.8%)

*Stage at treatment of NED patients *(*N* = 29)		*Stage at treatment of WED patients *(*N* = 20)	
Stage II	2 (6.9%)	Stage III (unresectable)	10 (50%)
Stage III (respectable)	22 (76%)	Stage IV: M1a	1 (5%)
Stage IV: M1b	2 (6.9%)	M1b	1 (5%)
M1c	3 (10.3%)	M1c	8 (40%)

*LDH values of NED patients*		*LDH values of WED patients*	
Time point 0:		Time point 0:	
normal	87% (20/23)	normal	78% (11/14)
above normal	13% (3/23)	above normal	21% (3/14)
Time point 1:		Time point 1:	
normal	78% (18/23)	normal	64% (7/11)
above normal	22% (5/23)	above normal	36% (4/11)

**Table 2:** sCEACAM1 correlates with S100B. ELISA measurements of posttreatment sCEACACM1 and S100B yielded values that were divided relative to median levels into “low” and “high.” The correlations between the two resulted “low” subgroups, as well as between the two “high” subgroups, were tested and found to be significant (*P* = 0.02). Percentages in each cubical refer to sCEACACM1 (first row) or to S100B (second row).

	Low S100B *N* = 25	High S100B *N* = 23
Low CEACAM1 *N* = 29	*N* = 19	*N* = 10
	65.5% (19/29)	34.5% (10/29)
	76% (19/25)	43.5% (10/23)

High CEACAM1 *N* = 19	*N* = 6	*N* = 13
	31.6% (6/19)	68.4% (13/19)
	24% (6/25)	56.5% (13/23)
